# Identification of a Common Non-Apoptotic Cell Death Mechanism in Hereditary Retinal Degeneration

**DOI:** 10.1371/journal.pone.0112142

**Published:** 2014-11-13

**Authors:** Blanca Arango-Gonzalez, Dragana Trifunović, Ayse Sahaboglu, Katharina Kranz, Stylianos Michalakis, Pietro Farinelli, Susanne Koch, Fred Koch, Sandra Cottet, Ulrike Janssen-Bienhold, Karin Dedek, Martin Biel, Eberhart Zrenner, Thomas Euler, Per Ekström, Marius Ueffing, François Paquet-Durand

**Affiliations:** 1 Institute for Ophthalmic Research, University of Tuebingen, Tuebingen, Germany; 2 Department of Neurobiology, University of Oldenburg, Oldenburg, Germany; 3 Center for Integrated Protein Science Munich and Department of Pharmacy - Center for Drug Research, Ludwig-Maximilians-University Munich, Munich, Germany; 4 Division of Ophthalmology, Department of Clinical Sciences, University of Lund, Lund, Sweden; 5 Institute for Research in Ophthalmology, Sion, Switzerland; 6 Centre for Integrative Neuroscience, University of Tuebingen, Tuebingen, Germany; Eye Hospital, Charité, Germany

## Abstract

Cell death in neurodegenerative diseases is often thought to be governed by apoptosis; however, an increasing body of evidence suggests the involvement of alternative cell death mechanisms in neuronal degeneration. We studied retinal neurodegeneration using 10 different animal models, covering all major groups of hereditary human blindness (*rd1*, *rd2*, *rd10*, *Cngb1* KO, *Rho* KO, S334ter, P23H, *Cnga3* KO, *cpfl1*, *Rpe65* KO), by investigating metabolic processes relevant for different forms of cell death. We show that apoptosis plays only a minor role in the inherited forms of retinal neurodegeneration studied, where instead, a non-apoptotic degenerative mechanism common to all mutants is of major importance. Hallmark features of this pathway are activation of histone deacetylase, poly-ADP-ribose-polymerase, and calpain, as well as accumulation of cyclic guanosine monophosphate and poly-ADP-ribose. Our work thus demonstrates the prevalence of alternative cell death mechanisms in inherited retinal degeneration and provides a rational basis for the design of mutation-independent treatments.

## Introduction

Apoptosis is a programmed cell death mechanism that is often invoked for neurodegenerative diseases. The classical apoptotic pathway starts with a BAX dependent permeabilisation of mitochondrial membranes, cytochrome c leakage to the cytoplasm and subsequent activation of initiator and executioner caspases [1]. Inherited neurodegenerative diseases of the retina are also generally thought to be governed by apoptotic cell death [2], [3], which has given rise to numerous attempts to use anti-apoptotic strategies for therapy development [4]–[6]. Unfortunately, these approaches were generally unsuccessful and efficient neuroprotective therapies for hereditary retinal degenerations (RD) such as retinitis pigmentosa (RP), Leber's congenital amaurosis (LCA), or Stargardt's disease are still missing. Recent findings suggest alternative, non-apoptotic cell death mechanisms for photoreceptor degeneration [7], [8]. Hence, we decided to systematically re-evaluate the situation in the retina using a variety of markers for both classical apoptosis and non-apoptotic cell death.

The retina harbours two general types of photoreceptors, rods, responsible for vision under dim-light conditions (*i.e.* at night), and cones, responsible for vision during bright daylight. In addition, the retina hosts a variety of different 2^nd^ and 3^rd^ order neurons, responsible for relaying photoreceptor output to the brain. For studies into hereditary degenerative mechanisms in the retina a large number of human homologous animal models are available [9], faithfully reproducing the photoreceptor degeneration phenotype. Two major categories of mutations and diseases can be distinguished: primary *rod* photoreceptor degeneration, which usually entails secondary cone death and complete blindness, and is characteristic of human diseases such as RP, LCA, or Usher syndrome. Primary *cone* photoreceptor degeneration, which leaves rods mostly unaffected but nevertheless causes a severe loss of visual acuity and daylight vision and typifies human diseases, such as cone-dystrophy, Stargardt's disease or age-related macular degeneration [10], [11].

**Figure 1:**
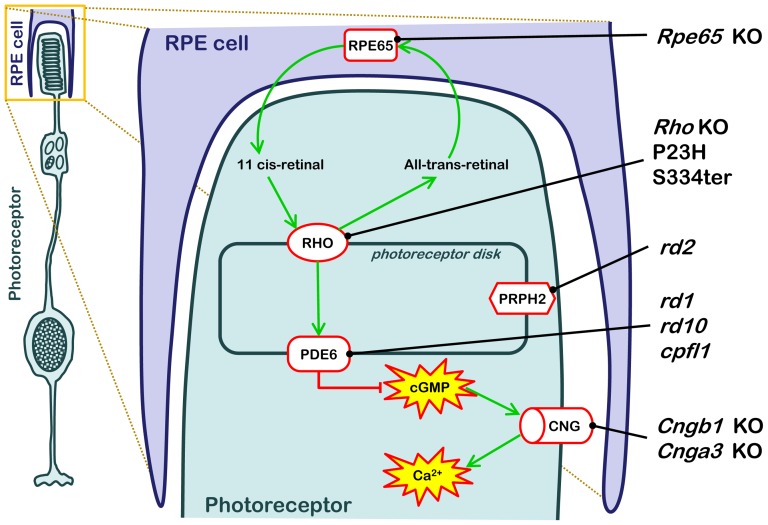
RD animal models used and their genetic defects. The cartoon illustrates the anatomical localization and metabolic consequences of the causative genetic mutations in the ten different RD animal models used in this study. RD causing mutations in these animal models interfere with the various stages of the phototransduction cascade, from the 11-cis-retinal recycling enzyme RPE65 (*Rpe65* KO), via the light-sensitive Rhodopsin (*Rho* KO, P23H, S334ter), cGMP-hydrolyzing phosphodiesterase-6 (PDE6; *rd1*, *rd10*, *cpfl1*), the structural protein Peripherin (*Prph2*; *rd2*), to the cyclic-nucleotide-gated (CNG; *Cngb1* KO, *Cnga3* KO) channel that allows for Ca^2+^-influx.

In the present study, we asked the question whether there was a common mechanism governing photoreceptor cell death independent of the initial causative genetic defect, since this could open up for broadly applicable therapies. To address the heterogeneity of hereditary photoreceptor degeneration, we employed ten different animal models RD (Figure 1), eight models for primary rod degeneration, as seen in autosomal dominant RP (P23H and S334ter transgenic rats) and autosomal recessive RP (*rd1*, *rd2*, *rd10*, *Cngb1* KO, *Rho* KO mice), as well as in LCA (*Rpe65* KO mice). In addition, we also included two animal models for primary cone death (*cpfl1*, *Cnga3* KO mice).

Surprisingly, our single cell resolution analysis of metabolic changes at the peak of cell death suggested that hereditary photoreceptor death was predominantly non-apoptotic, with only a marginal role, if any, for apoptosis. Instead, our study delineated a non-apoptotic cell death pathway and highlighted the general importance of this pathway for photoreceptor neurodegeneration. This finding has major ramifications for future therapy developments.

## Materials and Methods

### Animals

Animals were housed under standard white cyclic lighting, had free access to food and water, and were used irrespective of gender. Ten different mouse lines (C3H or C57Bl6 background) either wild-type or carrying naturally occurring mutations or engineered genetic deletions were used together with three different rat lines (CD background) expressing different rhodopsin transgenes (see Table 1). Day of birth was considered as postnatal day (P) 0. All procedures were approved by the respective local ethics and animal protection authorities and performed in compliance with the ARVO statement for the use of animals in Ophthalmic and Visual Research. Specifically, procedures performed in Tübingen (concerning C3H *wt*, C57Bl6 *wt*, *rd1*, *rd2*, *rd10*, *cpfl1*, CD *wt*, S334ter, and P23H animals) were reviewed and approved by the Tuebingen University “Einrichtung für Tierschutz, Tierärztlichen Dienst und Labortierkunde”. Procedures performed in Munich (on *Cngb1* KO and *Cnga3* KO animals) were reviewed and approved by the "Regierung von Oberbayern". Procedures performed in Oldenburg (on *Rho* KO animals) were reviewed and approved by the Oldenburg University animal welfare committee. Procedures performed in Sion (on *Rpe65* KO animals) were reviewed and approved by the Veterinary Service of the State of Valais (Switzerland). Procedures performed in Lund (*rd1*, *rd2* animals) adhered to permit # M220/09 issued by the local animal ethics committee. All efforts were made to minimize the number of animals used and their suffering.

**Table 1:** List of animals used, genes affected, and original references (where applicable).

Background/Line	Mutant	Gene	Reference
C3H	wild-type	-	[51]
C3H	*rd1*	*Pde6b*	[52]
C3H	*rd2*	*Prph2*	[53]
C57Bl/6J	wild-type	*-*	-
C57Bl/6	*Rho* KO	*Rho*	[54]
C57Bl/6N	*Cngb1* KO	*Cngb1*	[55]
C57Bl/6N	*Cnga3* KO	*Cnga3*	[56]
C57Bl/6J	*cpfl1*	*Pde6c*	[57]
C57Bl/6	*Rpe65* KO	*Rpe65*	[49]
C57Bl/6J	*rd10*	*Pde6b*	[58]
Crl: CD(SD)	wild-type	*-*	-
Crl: CD(SD)	P23H tg	*Rho*	[59]
Crl: CD(SD)	S334ter tg	*Rho*	[59]

Italic fonts indicate mutant name or affected gene.

### Histology, immunohistochemistry, and immunofluorescence

Animals were sacrificed in the morning (10–11 am), their eyes enucleated and fixed in 4% paraformaldehyde (PFA) in 0.1 M phosphate buffer (pH 7.4) for 45 min at 4°C. PFA fixation was followed by cryoprotection in graded sucrose solutions (10, 20, 30%). Unfixed eyecups were directly embedded in cryomatrix (Tissue-Tek, Leica, Bensheim, Germany). Sagittal 12 µm sections were obtained and stored at −20°C.

Sections were incubated overnight at 4°C with primary antibodies (Table 2). Immunostaining was performed employing the avidin-biotin-peroxidase technique (Vectastain ABC system, Vector laboratories, Burlingame, CA). Immunofluorescence was performed using Alexa Fluor 488-conjugated secondary antibodies (Molecular Probes, Inc. Eugene, USA). Negative controls were carried out by omitting the primary antibody. Sections were mounted with Vectashield (Vectorlabs, Burlingame, CA, USA) for imaging.

**Table 2:** List of antibodies used in this study.

Antigen	Source/Cat. Number	Dilution IF/IHC	Reference
BAX (clone 6A7)	Sigma/B8429	1∶20	[60]
Cleaved Caspase-3 (Asp175) (clone 5A1E)	Cell Signalling/9664	1∶300	[61]
Cleaved Caspase-9 (Asp353) (rabbit, polyclonal)	Abcam/ab52298	1∶100	[16]
Cytochrome C (clone 7H8.2C12)	Abcam/mab13575	1∶2000	[62]
cGMP (sheep, polyclonal)	Prof. Harry Steinbusch, Maastricht University, The Netherlands	1∶500	[63]
PAR (clone 10H)	Enzo/ALX-804-220	1∶200	[12]

### TUNEL Assay

Terminal deoxynucleotidyl transferase dUTP nick end labelling (TUNEL) assay was performed using an *in situ* cell death detection kit (Fluorescein or TMR; Roche Diagnostics GmbH, Mannheim, Germany). For controls terminal deoxynucleotidyl transferase enzyme was either omitted from the labelling solution (negative control), or sections were pre-treated for 30 min with DNAse I (Roche, 3 U/ml) in 50 mM Tris-HCl, pH 7.5, 1 mg/ml BSA to induce DNA strand breaks (positive control). While negative control gave no staining, positive control stained all nuclei in all layers of the retina [12].

### Calpain *in situ* activity assay

Calpain activity was investigated with an enzymatic *in situ* assay [13]. Briefly, unfixed cryosections were incubated for 15 min in calpain reaction buffer (CRB; 25 mM HEPES, 65 mM KCl, 2 mM MgCl^2^, 1,5 mM CaCl^2^, 2 mM DTT) and then incubated at 35°C for 1 h in the dark in CRB with 2 mM fluorescent calpain substrate 7-amino-4-chloromethylcoumarin, t-BOC-Leucyl-L-methionine amide (CMAC, t-BOC-Leu-Met; Molecular Probes, Inc. Eugene, USA). Fluorescence was uncaged by calpain-dependent cleavage of t-Boc-Leu-Met-CMAC.

### Poly-ADP-ribose polymerase (PARP) *in situ* activity assay

Unfixed cryosections were incubated in an avidin/biotin blocking kit (Vector Laboratories, Burlingame, USA), followed by incubation at 37°C for 2 h in PARP reaction mixture containing 10 mM MgCl_2_, 1 mM DTT, 5 µM biotinylated NAD^+^ (Trevigen, Gaithersburg, USA) in 100 mM Tris buffer with 0.2% Triton X-100 (pH 8.0). Biotin incorporation was detected by avidin - Alexa Fluor 488 conjugate (1∶800, 1 h at room temperature). For controls biotinylated NAD+ was omitted from the reaction mixture [14].

### HDAC *in situ* activity assay

HDAC activity assays were performed on retinal cryosections obtained from 4% PFA fixed eyes. Retinal sections were exposed to 200 µM Fluor de Lys-SIRT2 deacetylase substrate (Biomol, Hamburg, Germany) and 500 µM NAD+ (Biomol) in assay buffer (50 mM Tris/HCl, pH 8.0; 137 mM NaCl; 2.7 mM KCl; 1 mM MgCl2) and incubated for 2 h at 37°C. The tissue sections were then washed three times for 5 min in PBS and subsequently fixed in Methanol at −20°C, for 20 min. After refixation, the sections were washed once again for 5 min in PBS, then incubated in 1x Developer II (Biomol) in assay buffer and immediately coversliped and viewed under the microscope. The inclusion of either 100 µM TSA (Sigma, Steinheim, Germany) or 2 mM NAM (Sigma) in the assay allows to distinguish between HDAC activities coming from class I, II or IV (inhibited by TSA) or from class III (sirtuin-type HDACs, inhibited by NAM) [15].

### Microscopy, cell counting, and statistical analysis

Light and fluorescence microscopy were usually performed at room temperature on an Axio Imager Z.1 ApoTome Microscope, equipped with a Zeiss Axiocam MRm digital camera. Images were captured using Zeiss Axiovision 4.7 software; representative pictures were taken from central areas of the retina using a 20x/0,8 Zeiss Plan-APOCHROMAT objective. Adobe Photoshop CS3 (Adobe Systems Incorporated, San Jose, CA) was used for primary image processing.

For quantifications, pictures were captured on three entire sagittal sections for at least three different animals for each genotype and age using Mosaic mode of Axiovision 4.7 at 20x magnification. The average area occupied by a photoreceptor cell (*i.e.* cell size) for each genotype and age was determined by counting DAPI-stained nuclei in 9 different areas (50×50 µm) of the retina. The total number of photoreceptor cells was estimated by dividing the outer nuclear layer (ONL) area by this average cell size. The number of positively labelled cells in the ONL was counted manually. To be able to compare the various markers in the different genotypes, we considered cells as positively labelled only if they showed a strong staining of either the photoreceptor nuclei or perinuclear areas. Since some markers actually stained predominantly the photoreceptor inner and/or outer segments (*i.e.* BAX, cGMP in *Cngb1* KO retina) these may thus in the present study have been systematically underestimated. Values obtained are given as fraction of total cell number in ONL (*i.e.* as percentage) and expressed as mean ± standard error of the mean (SEM). For statistical comparisons the unpaired Student t-test as implemented in Prism 5 for Windows (GraphPad Software, La Jolla, CA) was employed.

## Results

### In RD models the peak of cell death varied depending on severity of genetic insult

**Figure 2:**
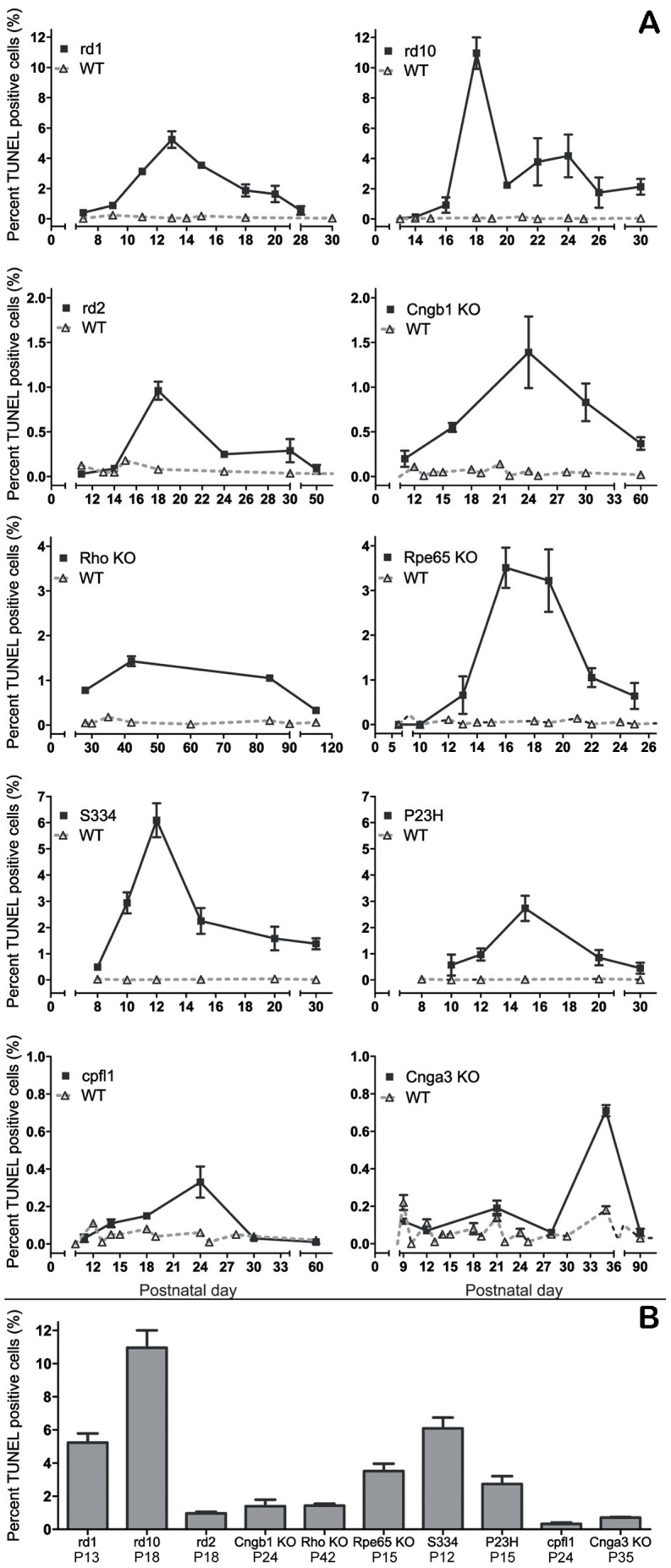
Progression of cell death in inherited RD models. Depending on the causative genetic insult, the temporal development of retinal degeneration is highly variable in the different animal models. The quantification of dying, TUNEL-positive photoreceptor cells in the outer nuclear layer (ONL) allowed determination of the evolution and the peak of photoreceptor death for each of these animal models (**A**). The peak was taken as reference point for the ensuing analysis of cell death mechanisms. The bar graph (**B**) shows a comparison of maximum peak heights for all ten RD models studied. Note the different scales in line graphs. Values are mean ± SEM from at least three different animals. See also Table S1 and S2.

To study the cell death mechanisms governing RD, we first performed a detailed analysis of the temporal progression of the degeneration for each of the 10 animal models used (Figure 1). We used the TUNEL assay to label dying cells at different postnatal ages and quantified the percentages of TUNEL-positive cells in the outer nuclear layer (ONL), *i.e.* the photoreceptor layer (Figure 2).

In all RD models, once the degeneration sets in, the TUNEL assay detected a moderate to strong elevation of dying cells when compared to the respective wild-type, depending on degeneration speed and whether rods or cones were affected. In each RD animal model the peak of cell death was identified (Figure 2) and all following experiments were performed at this time-point to increase the chances of detecting characteristic cell death processes. From previous experiments [12], [15]–[17], we know that the peak of TUNEL also corresponds to a strong activation of critical cell death processes; both for apoptotic and non-apoptotic cell death (*cf.* Figure S1). For the different animal models these time-points were: *rd1*  =  Postnatal day 13 (P13), *rd10*  =  P18, *rd2*  =  P18, *Cngb1* KO  =  P24, *Rho* KO  =  P42, *Rpe65* KO  =  P16, *cpfl1*  =  P24, *Cnga3* KO  =  P35, S334ter  =  P12, P23H  =  P15 (Data for *rd1*, *cpfl1*, S334ter, and P23H adapted from [16]–[18], respectively).

Since photoreceptor cell death is often seen as an apoptotic process [2], [3], we initially focused our analysis on detecting characteristic markers for apoptosis, and then extended our investigation to also include metabolic processes involved in alternative cell death mechanisms. To assess the extent to which apoptotic or non-apoptotic cell death mechanisms were active in the different animal models, we compared the number of cells displaying a specific metabolic activity with the number of TUNEL-positive cells in both mutant and wild-type retina (Table S1 and S2).

### Apoptosis was restricted to degenerating S334ter retina

**Figure 3:**
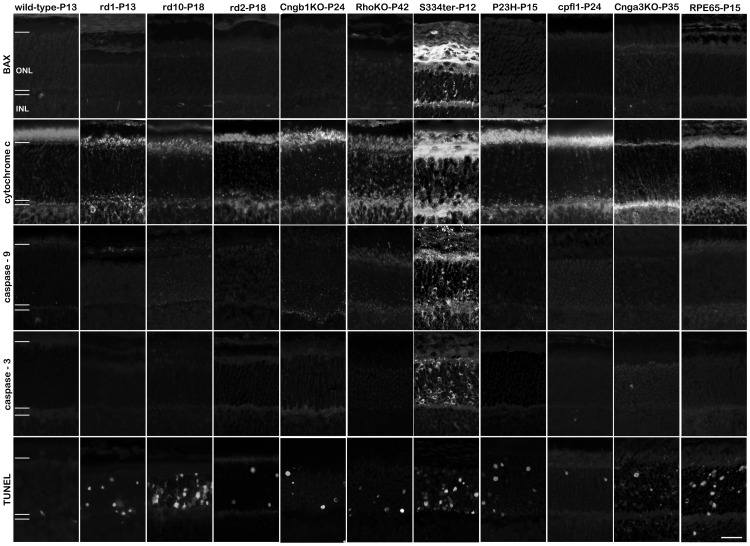
Apoptosis in the retina is restricted to the S334ter rat model. The analysis of BAX expression, mitochondrial cytochrome c release, activation of caspase-9 and -3 shows essentially no positive detection in 9 out of 10 animal models for hereditary retinal degeneration. The notable exception was the S334ter transgenic rat which harbours a mutation in the rhodopsin gene leading to a truncated protein and in which many photoreceptors were positive for apoptosis. In all other animal models, while there were cells displaying clear evidence for apoptosis, their numbers were within the wild-type levels, indicating that this was related to physiological, developmental cell death, which is characteristic for the postnatal rodent retina. Importantly, the numbers of apoptotic cells did not match the numbers of mutation-induced dying cells as evidenced by the TUNEL assay. Scale bar 20 µm.

We looked for increased expression, localization, or activation of Bcl-2–associated X protein (BAX), cytochrome c, cleaved, activated caspase-9 and -3 (Figure 3, quantification in Table S1 and S2). Increases in these apoptotic markers were found only in the S334ter model when compared to the corresponding wild-type.

Classical apoptosis starts with an activation of BAX [1]. Although early studies have already ruled out an involvement of BAX in RD [19], a recent study reported on the apparent activation of BAX in *rd1*, P23H, and Rho KO mice [20]. Nevertheless, in our hands a significant BAX activation (using the same antibody as in [20], Table 2) was observed only in S334ter retina. Here, prominent BAX staining was observed near mitochondria, in particular in individual photoreceptor inner segments, synaptic terminals, and occasionally around nuclei (Figure 3, Figure S2). This staining pattern in S334ter ONL is consistent with the reported role of BAX in the formation of the mitochondrial permeability transition pore [1].

Consequently, cytochrome c release from mitochondria was observed as an increased staining of individual photoreceptor cells in the S334ter ONL (Figure 3). A relative increase of cytochrome c leakage was found in *cpfl1* retina, however, this was not statistically significant (Table S1). Increased caspase-9 staining was present in S334ter retina only, with a peri-nuclear staining predominantly in the lower part of the ONL. A very similar staining pattern was found using an antibody specific for activated, cleaved caspase-3, again exclusively in S334ter retinal sections. These data are in line with previous studies [16], [21].

Thus, whereas large numbers of TUNEL-positive cells were detected in all analysed RD models, clear evidence for apoptosis was only detected in S334ter rats. This suggested the execution of alternative, non-apoptotic cell death mechanisms.

### Non-apoptotic cell death in photoreceptor degeneration

We have previously shown that rod photoreceptor degeneration in *rd1* mice is characterized by accumulation of cyclic guanosine monophosphate (cGMP), increased activities of histone deacetylases (HDAC), poly-ADP-ribose polymerases (PARP), and calpains [13], [15], [22].

**Figure 4:**
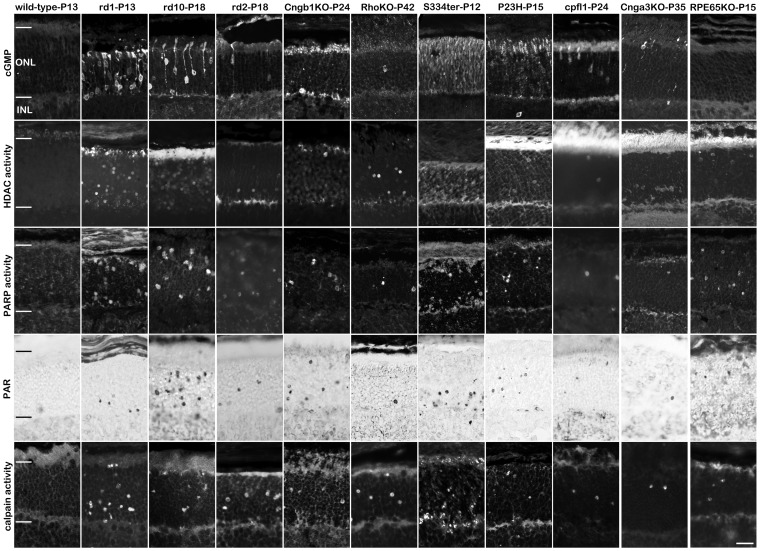
Cell death in hereditary retinal degeneration is predominantly non-apoptotic. In 10 out of 10 animal models for hereditary retinal degeneration, large numbers of photoreceptors display cGMP accumulation, HDAC and PARP activity, PAR accumulation, and calpain activity, respectively. Intriguingly, these non-apoptotic markers are prominent even in the S334ter retina, concomitant with this also showing signs of apoptosis. This suggests that in S334ter retina two different cell death mechanisms may run in parallel while in all other studied RD models the mutation-induced cell death followed a non-apoptotic mechanism. Scale bar 20 µm.

cGMP accumulation in phosphodiesterase-6 mutants (*rd1*, *rd10*, *cpfl1*) is a direct consequence of the lack of phosphodiesterase activity that normally hydrolyses cGMP. Surprisingly, significant cGMP accumulation was observed also in all other analysed mouse and rat models (Figure 4, Table S1) except for *Rpe65* KO retina, where the initial causative defect does not reside in photoreceptors themselves but in retinal pigment epithelial cells. However, the patterns of cGMP accumulation varied between different RD models (Figure 4). In the case of *rd1*, *rd10*, *rd2*, *Cnga3* and *cpfl1* cGMP was visible in cell bodies as well as in photoreceptor inner/outer segments, whereas in *Cngb1* KO retina the signal was more prominent in inner/outer segments. For methodological reasons, we only quantified cGMP positive cell bodies. As a consequence, most likely the true number of photoreceptors showing elevated cGMP levels is higher in *Cngb1* KO retina than assessed here. P23H and S334ter rat retinas were characterized by diffuse cGMP accumulation in the ONL, contrary to *Rho* KO mice in which only very few nuclei were cGMP-positive.

The HDAC assay revealed significantly increased activity in all the analysed mutants when compared to corresponding wild-type (Figure 4). The number of nuclei stained with the HDAC assay varied between different mutants (Table S1 and S2) with more cells showing HDAC activity in the case of *rd1*, *rd10*, and S334ter and less positive cells in the case of *Cngb1* KO and *Rpe65* KO.

To determine if poly-ADP-ribosylation, as an additional epigenetic process, was involved in photoreceptor degeneration, we looked for increased PARP *in situ* activity as well as for accumulation of poly-ADP-ribosylated proteins (PAR), *i.e.* the products of PARP activity. Nuclear staining of both PARP activity and PAR followed the patterns observed for HDAC activity. Mutants characterized by a high number of TUNEL-positive cells (*rd1*, *rd10*, S334ter and *Rpe65* KO) also displayed comparatively higher numbers of both PARP and PAR stained cells compared to models with low degeneration rates. The *in situ* staining for calpain activity was also significantly increased in all analysed RD models (Figure 4, quantification in Table S1).

**Figure 5:**
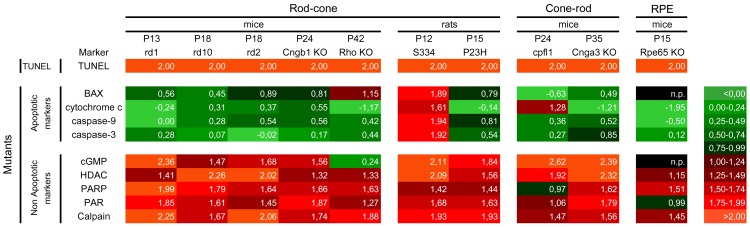
Heat map representing metabolic activities in different RD models. The RD models were grouped according to the peak of degeneration, the cell type affected by the mutation (rod, cone, RPE), and species (mouse, rat). The number of TUNEL-positive cells in each model was normalized to 100, expressed as logarithm, and compared with the number of positively labelled cells for each marker. The heat map clearly illustrates the prevalence of non-apoptotic *vs*. apoptotic cell death in 9 out of 10 RD models. The S334ter rhodopsin mutant was unique, showing concurrent activation of both cell death pathways. n.p.: null positive. See also Table S1 and S2.

To compare the different cell death processes, we related the numbers of positive cells detected by each individual assay to the numbers of TUNEL positive cells. To match the various RD models and their very different degeneration kinetics with each other, all values were expressed as logarithm to base 10. Since the TUNEL values were defined as 100%, its logarithm was 2. This comparative analysis highlighted the fact that non-apoptotic processes were clearly dominant for photoreceptor degeneration in all RD models (Figure 5). This was also true for the S334ter model which, interestingly, showed the additional involvement of apoptotic cell death. We also analysed the relative contribution of apoptotic and non-apoptotic processes to developmental cell death in wild-type retina (P13-P42). Here, the relative contributions of apoptotic and non-apoptotic cell death mechanisms appeared to be equally important (Figure S3).

## Discussion

Our study provides a detailed and comprehensive overview of the temporal progression and the kinetics of cell death in ten different, commonly used RD animal models. These RD models harbour genetic defects mostly affecting the phototransduction cascade but include also such which are disturbing the visual cycle (*Rpe65* KO) and the structural integrity of the outer segment (*rd2*). As a result, the comparative analysis of characteristic cell death processes for the first time highlights the over-riding importance of a common, alternative mechanism for photoreceptor degeneration. Contrary to previous studies on retinal degeneration mechanisms [23], [24] our study focused on the elevated activity and presence of key enzymes and/or metabolites, respectively, and thus may be seen as a first attempt to assess the so called reactome or metabolome (www.reactome.org; [25]) of photoreceptor degeneration at the level of the individual dying cell.

To put our report in a perspective, many studies on cell death in the retina and other parts of the central nervous system have previously resorted to tissue based methods (*e.g.* micro-array, western blot) [23], [24]. Such methods are particularly useful in conditions where there is a homogenous cell population and a highly synchronized onset of cell death and are thus ideal, for instance, for cell culture. However, in a complex neuronal tissue such as the retina, with>50 different neuronal cell types among which only one – the rod photoreceptor – undergoes non-synchronized primary degeneration, with cell death of individual photoreceptors spread out over a time of weeks to many years, tissue based analysis runs the risk of suffering from very low detection rates and overall poor signal-to-noise ratio. For our analysis, we thus focussed on methods that afforded cellular resolution to be able to unequivocally attribute cell death related processes to primary photoreceptor death and to distinguish these processes from secondary or tertiary events.

### Apoptosis during retinal degeneration

Previous studies on cell death in hereditary retinal degeneration have often suggested apoptosis as the main degenerative mechanism [2], [3], [26]. These earlier studies, however, based their conclusion on analysis methods now known not to discriminate between apoptosis and other forms of cell death. For instance, the TUNEL assay, originally thought to be a marker for apoptosis [27], generally labels all kinds of dying cells, including necrotic cells [28].

Apoptosis may be defined as an active process resulting in orderly self-disintegration of a cell. Hallmark features of apoptosis include an up-regulation of pro-apoptotic genes and proteins, such as the transcription factor c-fos and in particular Bcl-2 family proteins such as BAX, which participate in forming the mitochondrial permeability transition pore (MPTP), allowing mitochondrial proteins including cytochrome c to enter the cytoplasm. Cytoplasmic cytochrome c aggregates with apoptotic protease-activating factor (APAF) and caspase-9 to form a multimeric protein complex termed the apoptosome [1]. This complex then cleaves and activates down-stream executioner caspases such as caspase-3.

Classical apoptosis occurs during retinal development until about 3–4 weeks post-natal [29]. Indeed, developmental apoptosis temporally coincides, at least partially, with mutation-induced cell death [7]. This introduces a confounding factor which may explain some of the contradictory reports in the literature. Our study demonstrates that wild-type photoreceptors are capable of executing apoptosis at least until P42; by contrast, however, we see that mutant photoreceptors normally take a non-apoptotic route as a means for orderly self-destruction.

Importantly, therapeutic strategies based on the inhibition of the apoptotic cascade have had little success or produced conflicting findings. For instance, neither the pharmacological inhibition of the caspase cascade [5], nor the genetic manipulation of Bcl-2 and Bcl-XL [30], c-fos [31], or caspase-3 [6] promoted long-term photoreceptor survival. On the other hand BAX KO may delay rod but not cone death in the *Rpe65* KO animals [4].

Recently, increased BAX activation was suggested to be connected to retinal degeneration in *rd1*, *Rho* KO, and P23H mice [20]. At present it is not clear whether these findings relate in part to developmental cell death (see above) or would have been interpreted differently if the study [20] had also included observations of a model with a much stronger BAX response, such as the S334ter rat investigated by us. At any rate, our results do not show any evidence for major BAX activation in degenerating retina, with the notable exception of S334ter photoreceptors. This model thus constitutes a “positive control” for BAX and further apoptotic markers, lending additional credit to our findings in all other mutants.

### Alternative cell death mechanisms

In recent years a growing body of evidence has suggested the activity of alternative cell death mechanisms in RD [8], [32]–[34]. The analysis of such mechanisms faces the major obstacle of identifying alternative and causative metabolic processes. In a number of previous studies, we showed activation of the cGMP targets protein kinase G (PKG) and cyclic nucleotide-gated (CNG) channel [22], [35] in degenerating *rd1* photoreceptors. Excessive cGMP signalling was associated with a strong increase in enzymatic activities of calpain-type proteases [13], PARP [12], and HDAC [15], which we found to be causally involved in photoreceptor cell death. Calpain activation, which was also seen by others in different RD models [20], is a well-established phenomenon in necrosis and alternative cell death mechanisms [21], [36]. While HDAC and PARP enzymes are ubiquitously expressed and involved in epigenetic gene regulation and DNA repair [37], respectively, their excessive activation has repeatedly been connected to alternative mechanisms of neuronal cell death [38]–[40].

We found that all these processes were also involved in RD caused by the different mutations, in various genes and in both mouse and rat. Importantly, the cellular resolution afforded by the used assays allowed clear distinction between cells dying an apoptotic death and cells dying through an alternative pathway. In this alternative pathway the activities of calpain and PARP activity co-localize to a large extent with the TUNEL assay [12], [17], while cGMP detection and HDAC activity do not [15], [41]. This could suggest that the latter two relate to early metabolic processes in the execution of cell death.

**Figure 6:**
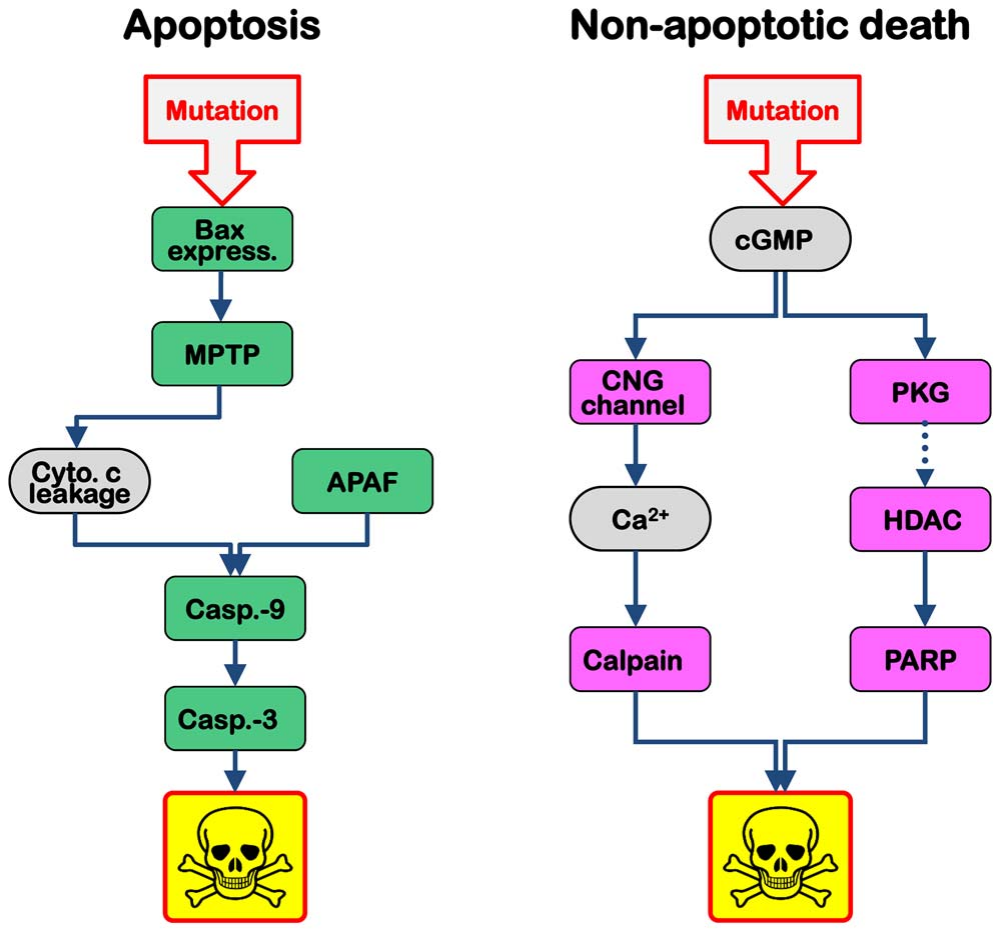
Two routes to cell death. Classical apoptosis, such as it occurs in S334ter transgenic photoreceptors, involves a mutation-induced up-regulation and translocation of BAX protein to form the mitochondrial permeability transition pore (MPTP). This leads to leakage of cytochrome c from the mitochondria to the cytoplasm, where it combines with apoptotic protease activating factor (APAF) and caspase-9 to form the apoptosome, which in turn activates down-stream executioner caspases, including caspase-3. In 9/10 RD animal models investigated here, photoreceptor death followed a different route: mutation-induced up-regulation of cGMP on the one hand causes activation of the CNG channel, leading to Ca2+ influx and calpain activation. On the other hand cGMP-dependent activation of protein kinase G (PKG) is associated with histone deacetylase (HDAC) and poly-ADP-ribose-polymerase (PARP) activation. Importantly, this alternative, non-apoptotic cell death mechanism offers a number of novel targets for neuroprotection of photoreceptors.

Together with other earlier data [8], [16], [42], [43] our present findings prompt us to propose a potential pathway for cGMP-induced cell death: Elevated levels of cGMP activate CNG channels and/or PKG to cause excessive Ca^2+^-influx and protein phosphorylation, respectively. As a possible consequence of the latter, PKG dependent phosphorylation could trigger HDAC activation [44], down-stream of which PARP can be activated [15]. Ca^2+^-influx might on the other hand, and in parallel, cause calpain activation [13], [35]. Both routes (Figure 6) act in unison to drive a photoreceptor cell to its demise, but, surprisingly, this alternative form of cGMP-induced cell death appears to be 4–6 times slower than apoptosis [41]. Importantly, the presence of this pathway and the connections between the different metabolic processes were confirmed by interventional experiments in the *rd1* mouse demonstrating the neuroprotective effects of inhibition of PKG [22], calpain [13], PARP [12], and HDAC [15].

The observed PARP activity deserves some additional considerations: In classical apoptosis the PARP enzyme is cleaved and inactivated by caspases, resulting in a specific 85 kDa PARP fragment, the presence of which is often used to characterize apoptosis as such [45]. In our study, we used two independent methods – immunostaining for the PARP activity product PAR and direct *in situ* PARP activity detection based on incorporation of NAD^+^ – to demonstrate PARP over-activation. Hence, what we found in mutant photoreceptors is the exact opposite of what would happen in apoptosis, which thus provides further evidence for a non-apoptotic photoreceptor cell death, an alternative cell death mechanism that could share some features with PARthanatos [40].

The fact that photoreceptors use a non-apoptotic mechanism when in principle they are capable of executing apoptosis raises the question as to what the physiological and even evolutionary advantage of this mechanism may be. Apoptosis is a process that requires energy in the form of ATP [1]. The insult caused by a genetic mutation may exhaust such energy resources to the point that apoptosis can no longer be executed. Necrosis on the other hand would result in inflammation and could cause additional extensive tissue damage. Hence, it may make sense for a cell to execute the slow, alternative and probably ATP-independent pathway laid out here to limit the damage to the surrounding neuronal tissue.

### Perspectives for mutation-independent RD treatment

An important consequence of the high genetic heterogeneity of retinal degenerations is that for any pathogenic mutation there may be only a very low number of patients [10], [11]. This calls for the development of mutation-independent treatments that could address larger groups of RD patients. The finding that the same non-apoptotic mechanism was the prevalent mode of cell death in 9/10 RD models strongly increases the chances to find neuroprotective treatments that are independent of the initial causal mutation. In the context of rare retinal diseases, such treatments appropriate for a large number of patients may dramatically improve the perspectives for both a successful clinical translation and the commercial viability of corresponding drugs.

We found that the alternative cell death mechanism described above was active in all investigated animal models. Of particular importance for this mechanism may be the observed accumulation of cGMP in mutant photoreceptors. While this was already known for retina suffering from mutations in *Pde6b* and *Pde6c* (*i.e. rd1*, *cpfl1*; [18], [46]), *Prph2* (i.e.*rd2* [22]), *Cngb1* and *Cnga3* [35], [42], our work also showed cGMP accumulation in retina suffering for three different types of rhodopsin mutations (*Rho* KO, S334ter, P23H). A potential explanation for this remarkable phenomenon in rhodopsin mutants could be either the longer life-times of activated rhodopsin resulting in a stimulation of cGMP synthesis and an increase in net cGMP [47] or a failure to activate downstream PDE6 in cases where rhodopsin is absent (*i.e*. in *Rho* KO).

While these findings highlight cGMP-signalling for the development of novel neuroprotective treatments, there is one exception: in *Rpe65* KO retina, we did not find elevations of cGMP. Indeed, here, unliganded opsin was proposed to cause a constitutive activation of phototransduction and hence low cGMP-levels [48]. On the other hand, since all further down-stream processes appear to be the same in all mutants investigated, a disruption of the visual cycle by *Rpe65* KO [49] might cause minor elevations of cGMP – perhaps below the detection levels of our immunohistological methods – and still trigger cell death.

Mutations in the same gene may potentially trigger distinct degenerative processes [16]. Our study more extensively shows how intragenic variability of RD mutations may initiate different cell death mechanisms: The recessive *rd1* and *rd10* mutations in the *Pde6b* gene result in activation of the same non-apoptotic pathways. This is also true for the recessive *Rho* KO and the dominant P23H mutation, but not for the dominant S334ter mutation. While all three mutations reside in the rhodopsin gene, the concurrent activation of apoptotic and non-apoptotic cell death observed in the S334ter situation suggests that human patients with similar mutations may need combination therapy targeting both degenerative pathways simultaneously. Likewise, since we found that photoreceptors (wild-type) are in principle able to execute apoptosis, we cannot exclude the possibility that under circumstances in which non-apoptotic cell death is blocked, the cell may switch to apoptosis. This possibility needs further investigation and might also require the development of combination therapies.

Another question, that will be important to address in the future, relates to the fact that all mutant photoreceptors carry a genetic defect that will eventually destroy them. Yet, the time-point at which a mutant photoreceptor dies appears to be entirely random, and, in the human situation, the time from the first to the last photoreceptors' death may cover many decades [10]. The exact reasons for this phenomenon are unknown but could be explained by stochastic effects similar to what is seen in the decay of radioactive elements [50]. This opens the possibility that even a minor shift in the dynamics of these stochastic processes – such as interference with processes like those studied here – could improve photoreceptor survival dramatically.

In conclusion, this work demonstrates the existence of a common, non-apoptotic cell death mechanism for hereditary photoreceptor degeneration. The tentative cell death pathway laid out here (Figure 6) provides a number of novel targets for neuroprotective treatment approaches [12], [13], [15], [16], [22] and, importantly, a unifying principle for RD caused by a variety of different mutations in different genes. As such, this common cell death pathway may be of major importance for future RD therapy developments and possibly for also other neurodegenerative diseases.

## Supporting Information

Figure S1 Correlation of selected cell death markers to loss of photoreceptors, related to Figure 1. Percentage of labelled ONLcells (left y-axis) and number of surviving photoreceptor rows (right y-axis) for (A) *rd1* mice, (B) P23H, and (C) S334ter transgenic rats. In all three models, calpain activation peaked together with the TUNEL assay, and correlated with the strongest loss in the number of photoreceptor rows. The grey area indicates the loss of photoreceptors. Throughout the retinal degeneration, activation of caspase-3 was ***absent*** in *rd1* and P23H retina, but ***present*** in S334ter retina. Values are mean from at least three different animals.
(TIF)

Figure S2 Expression of activated BAX in wild-type, rd1 and S334ter retina. In wild-type mouse retina at P11 (left panel), a mouse monoclonal antibody directed against activated BAX (clone 6A7) detected positive cells only rarely, but then in all layers of the retina. The white arrowhead indicates a cell positive for activated BAX in the ganglion cell layer (GCL). In *rd1* mouse retina at P11 – the onset of RD in this model – activated BAX is detected only very rarely, with BAX detection levels very similar to age-matched wild-type (middle panel; cf. Table S2). In contrast to this, in the outer nuclear layer (ONL) of P12 S334ter rat retina, the BAX antibody immunodecorates mitochondria, in particular in individual photoreceptor inner segments, synaptic terminals, and perinuclear areas (right panel). This mitochondria specific staining pattern in S334ter retina is consistent with the reported role of BAX in the formation of the mitochondrial permeability transition pore and the initiation of apoptosis. Images are representative for immunostainings obtained from at least three different animals for each genotype. Note that use of secondary anti-mouse antibodies led to an unspecific IGG decoration in inner retinal blood vessels in mouse tissues (see asterisks in wild-type, *rd1*). INL  =  inner nuclear layer.
(TIF)

Figure S3 Cell death markers in wild-type mouse retina. Well-type retina occasionally showed cells positive for both apoptotic and non-apoptotic cell death markers (A). As the number of positive cells is rather small, please note that the pictures shown are selected not as the representative but somewhat an exaggeration of the real number of dying cells. Heat map representing metabolic activities in corresponding wild-types (B), similarly as in Figure 5 for RD mutants, shows that cell death during wild-type retina development displayed activation of both apoptotic and non-apoptotic pathways. Scale bar 20 µm. n.p.: null positive. See also Table S2.
(TIF)

Table S1 Quantification of cell death processes in 10 different RD animals related to Figures 1 and 4. Numbers given represent mean values for the percentages of positive cells for each marker, followed by standard error of the mean (SEM), and p-values for comparisons with corresponding, age-matched WT. Green label indicates statistically significant p-values (p<0.05); red label indicates non-significance. Significant differences between RD mutants and WT were found almost only for non-apoptotic processes, with the notable exception of the S334ter mutant where also apoptotic processes were significantly activated. Note that in contrast to Fig. 4, here, values were not normalized to the numbers of TUNEL positive, dying cells.
(TIF)

Table S2 Quantification of labelled photoreceptors in different RD models related to Figures 1 and 4. For each genotype, at the respective peak of degeneration, the percentage of cells positively labelled for the various cell death processes is given as mean value, followed by SEM, and number (n) of different specimens analysed. To assess the relative importance of these processes for retinal degeneration the percentage of TUNEL positive cells is also given.
(TIF)
