# Supplementary figures and images for: Identification of a Common Non-Apoptotic Cell Death Mechanism in Hereditary Retinal Degeneration
Source: PLoS One. 2014 Nov 13;9(11):e112142. doi: 10.1371/journal.pone.0112142 (PMC4230983; doi:10.1371/journal.pone.0112142)

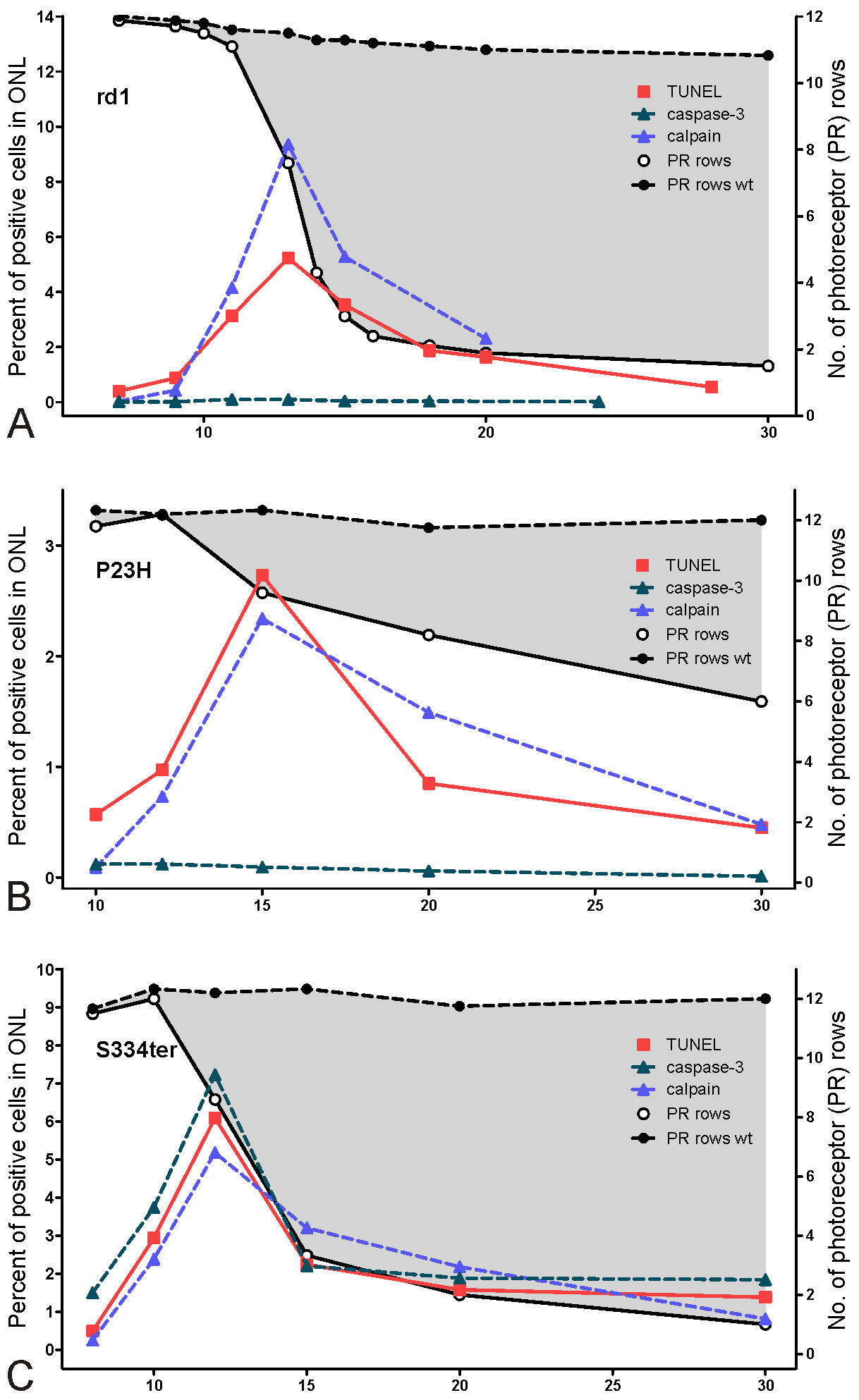

Supplement: Figure S1 — Correlation of selected cell death markers to loss of photoreceptors, related to Figure 1. Percentage of labelled ONLcells (left y-axis) and number of surviving photoreceptor rows (right y-axis) for (A) rd1 mice, (B) P23H, and (C) S334ter transgenic rats. In all three models, calpain activation peaked together with the TUNEL assay, and correlated with the strongest loss in the number of photoreceptor rows. The grey area indicates the loss of photoreceptors. Throughout the retinal degeneration, activation of caspase-3 was absent in rd1 and P23H retina, but present in S334ter retina. Values are mean from at least three different animals. (TIF) [file pone.0112142.s001.tif]

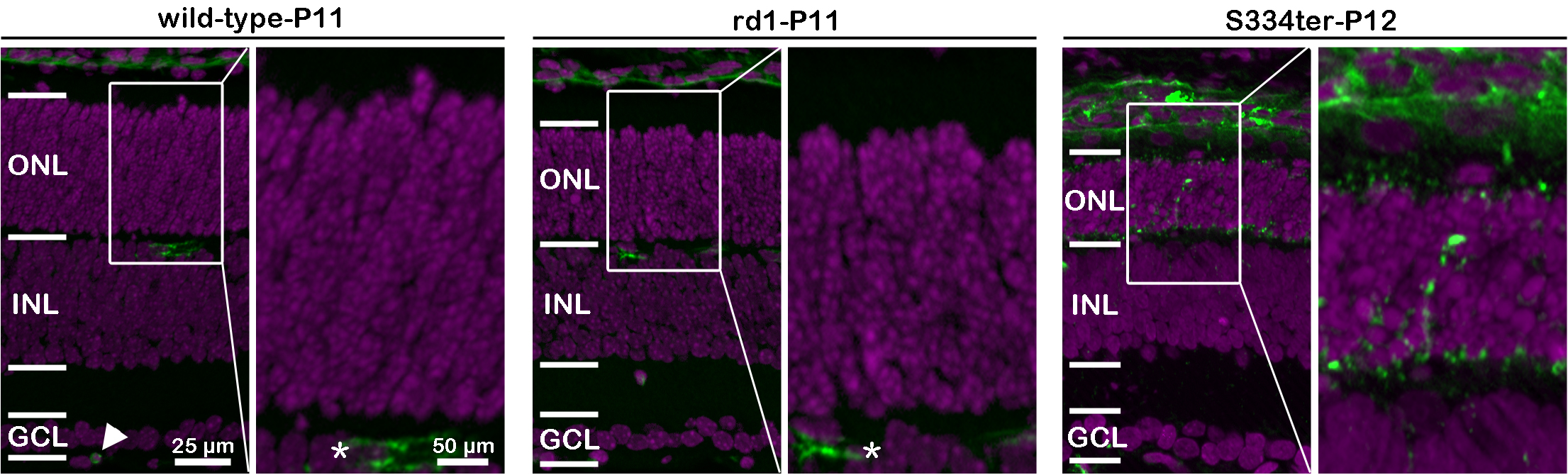

Supplement: Figure S2 — Expression of activated BAX in wild-type, rd1 and S334ter retina. In wild-type mouse retina at P11 (left panel), a mouse monoclonal antibody directed against activated BAX (clone 6A7) detected positive cells only rarely, but then in all layers of the retina. The white arrowhead indicates a cell positive for activated BAX in the ganglion cell layer (GCL). In rd1 mouse retina at P11 – the onset of RD in this model – activated BAX is detected only very rarely, with BAX detection levels very similar to age-matched wild-type (middle panel; cf. Table S2). In contrast to this, in the outer nuclear layer (ONL) of P12 S334ter rat retina, the BAX antibody immunodecorates mitochondria, in particular in individual photoreceptor inner segments, synaptic terminals, and perinuclear areas (right panel). This mitochondria specific staining pattern in S334ter retina is consistent with the reported role of BAX in the formation of the mitochondrial permeability transition pore and the initiation of apoptosis. Images are representative for immunostainings obtained from at least three different animals for each genotype. Note that use of secondary anti-mouse antibodies led to an unspecific IGG decoration in inner retinal blood vessels in mouse tissues (see asterisks in wild-type, rd1). INL = inner nuclear layer. (TIF) [file pone.0112142.s002.tif]

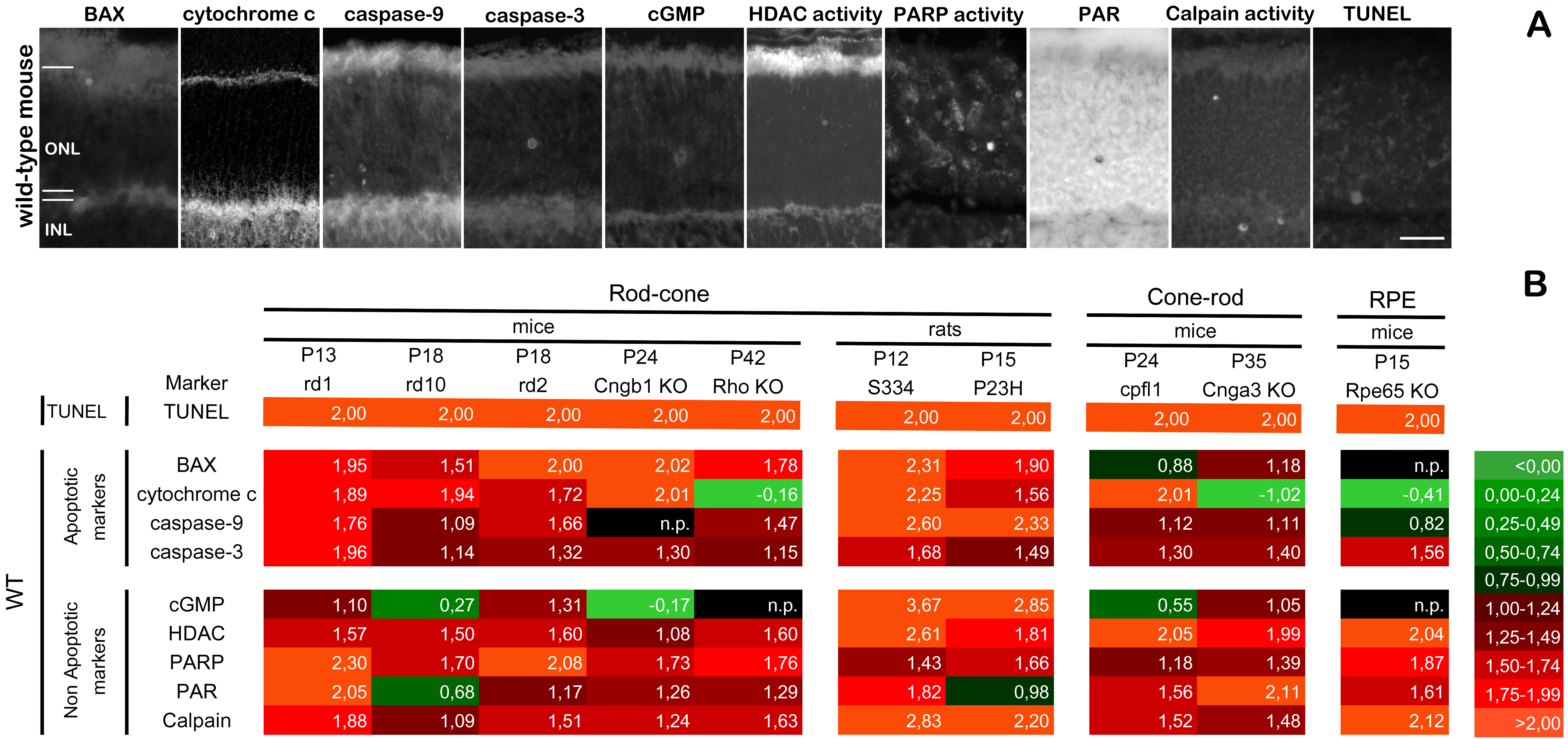

Supplement: Figure S3 — Cell death markers in wild-type mouse retina. Well-type retina occasionally showed cells positive for both apoptotic and non-apoptotic cell death markers (A). As the number of positive cells is rather small, please note that the pictures shown are selected not as the representative but somewhat an exaggeration of the real number of dying cells. Heat map representing metabolic activities in corresponding wild-types (B), similarly as in Figure 5 for RD mutants, shows that cell death during wild-type retina development displayed activation of both apoptotic and non-apoptotic pathways. Scale bar 20 µm. n.p.: null positive. See also Table S2. (TIF) [file pone.0112142.s003.tif]
